# Oral health-related quality of life in implant-supported rehabilitations: a prospective single-center observational cohort study

**DOI:** 10.1186/s12903-024-04265-y

**Published:** 2024-05-04

**Authors:** Mattia Manfredini, Matteo Pellegrini, Marta Rigoni, Valentina Veronesi, Mario Beretta, Carlo Maiorana, Pier Paolo Poli

**Affiliations:** 1 Department of Biomedical, Surgical and Dental Sciences, University of Milan, Via della Commenda 10, 20122 Milan, Italy; 2 Implant Center for Edentulism and Jawbone Atrophies, Maxillo-Facial Surgery and Dental Unit, Fondazione IRCCS Ca’ Granda Ospedale Maggiore Policlinico, Via Commenda 10, 20122 Milan, Italy

**Keywords:** Dental implants, Dentistry, Implant-supported rehabilitations, Oral health, Psychosocial impact, Quality of life.

## Abstract

**Background:**

Oral Health-Related Quality of Life (OHRQoL) is a comprehensive concept covering daily comfort, self-esteem, and satisfaction with oral health, including functional, psychological, and social aspects, as well as pain experiences. Despite abundant research on OHRQoL related to oral diseases and hygiene, there is limited data on how patients perceive changes after implant-prosthetic rehabilitation. This study aimed to evaluate OHRQoL and aesthetic perception using OHIP-14 and VAS scales respectively, before (baseline-TB), during (provisional prostheses-TP), and after (definitive prostheses-TD) implant-prosthetic rehabilitation. It also explored the impact of biological sex, substitution numbers, and aesthetic interventions on OHRQoL and VAS scores, along with changes in OHIP-14 domains.

**Methods:**

A longitudinal prospective single-center observational cohort study was conducted with patients requiring implant-prosthetic rehabilitation. Quality of life relating to dental implants was assessed through the Italian version of Oral Health Impact Profile-14 (IOHIP-14), which has a summary score from 14 to 70. Patients’ perceived aesthetic was analyzed through a VAS scale from 0 to 100. Generalized Linear Mixed Effect Models, Linear Mixed Effect Models, and Friedman test analyzed patient responses.

**Results:**

99 patients (35 males, 64 females) aged 61–74, receiving various prosthetic interventions, were enrolled. Both provisional and definitive prosthetic interventions significantly decreased the odds of a worse quality of life compared to baseline, with odds ratios of 0.04 and 0.01 respectively. VAS scores increased significantly after both interventions, with estimated increases of 30.44 and 51.97 points respectively. Patient-level variability was notable, with an Intraclass Correlation Coefficient (ICC) of 0.43. While biological sex, substitution numbers, and aesthetic interventions didn’t significantly affect VAS scores, OHRQoL domains showed significant changes post-intervention.

**Conclusions:**

These findings support the effectiveness of implant-prosthetic interventions in improving the quality of life and perceived aesthetics of patients undergoing oral rehabilitation. They have important implications for clinical practice, highlighting the importance of individualized treatment approaches to optimize patient outcomes and satisfaction in oral health care.

**Supplementary Information:**

The online version contains supplementary material available at 10.1186/s12903-024-04265-y.

## Background

Oral health-related quality of life (OHRQoL) assessment related to implant-prosthetic rehabilitation is a phenomenon that has emerged since the early 2000s [1]. Slade [2] identified the change in health perception from the simple absence of disease and infirmity to complete physical, mental, and social well-being, echoing the original World Health Organization (WHO) definition [3]. This change took place in the second half of the 20th century and was assessed by WHO as the key issue in the conception of Health-Related Quality of Life (HRQoL) and later OHRQoL, as a “silent revolution” in the values of industrialized societies from materialistic values focusing on economic stability and security to values centered on self-determination and self-actualization [4].

In the 1970s, Davis [5] stated how, apart from pain and life-threatening cancers, other oral diseases have no impact on social life, being related only to cosmetic problems.

Subsequently, the concept of OHRQoL began to evolve. There was growing evidence that oral diseases could also have a significant impact on social roles. The clinical indicators used in diagnosing and monitoring oral diseases such as dental caries or periodontal disease were not entirely adequate to capture the new concept of health declared by WHO, particularly aspects of mental and social well-being [6–9]. As a result, researchers began to develop alternative methods, particularly patient-completed questionnaires, that would assess the physical, psychological, and social impact of oral conditions on an individual [10].

Thus, the OHRQoL becomes “a multidimensional construct” that reflects people’s comfort when eating, sleeping, and engaging in social interactions, their self-esteem, as well as their satisfaction concerning oral health [11]. OHRQoL is associated with functional factors, psychological factors, social factors, and experience of pain or discomfort [12].

Information on quality of life makes it possible to assess feelings and perceptions at the individual level, increasing opportunities for communication between professionals and patients, improving understanding of the impact of oral health on the subject’s and family’s lives, and measuring the clinical outcomes of the interventions performed [13].

In the scientific literature, to truly define OHRQoL, many questionnaires have been created to quantitatively assess the actual improvement of quality of life about oral health. To this end, the European Commission suggests using the Oral Health Impact Profile (OHIP) as a tool to assess OHRQoL, as it has been well designed, extensively tested, has longitudinal and discriminative validity, and focuses on psychological and behavioral issues [14]. The original extended version (OHIP-49) contained 49 items and was based on a conceptual framework regarding oral health and its functional and psychological consequences [15]. A reduced questionnaire was designed to simplify the original version: the OHIP-14 [16]. The latter is simple to use, tested with positive results for psychometric qualities (validity and reliability) in several studies and different populations, sensitive to the measurement of clinical effects of treatment, with measurement properties comparable to the OHIP-49 [16].

The evaluation of OHRQoL in implant rehabilitation is important for several reasons [17, 18]: (1) patient orientation, implant rehabilitation aims to enhance the functionality and aesthetics of the patient (the assessment of OHRQoL involves the patient’s perspective, enabling an understanding of their views on oral health and overall well-being. This aids in tailoring the treatment plan based on the patient’s needs and expectations); (2) measurement of psychosocial impact, dental implants not only affect masticatory function but also impact the patient’s aesthetic appearance and self-confidence (the evaluation of OHRQoL allows for the measurement of the psychosocial effects of implant rehabilitation, including aspects such as self-esteem, social interaction, and overall satisfaction); (3) treatment efficiency evaluation, OHRQoL can serve as an indicator of the effectiveness of implant treatment (measuring the change in oral health-related quality of life before and after treatment provides crucial information on the success of the procedure and patient satisfaction); (4) informed clinical decision-making, the assessment of OHRQoL can assist oral health professionals in making informed clinical decisions (understanding the treatment’s impact on the patient’s quality of life helps formulate more accurate and tailored treatment plans based on the individual’s specific needs); (5) patient-dentist communication, discussing OHRQoL facilitates communication between the patient and oral health professionals, establishing realistic expectations regarding treatment outcomes (it enables the patient to be more involved in decisions related to their oral health).

Analyzing the current success criteria applicable in the assessment of implant-prosthetic rehabilitation, in addition to clinical criteria such as implant integration, absence of pain or discomfort, effective and comfortable chewing, gingival health, long-term implant stability, and maintenance of the surrounding bone structure over time, criteria such as aesthetic appearance and patient satisfaction are key and equally important criteria alongside clinical criteria [19–21].

Furthermore, considering the importance of aesthetic evaluation, two methods described in the literature are utilized for its assessment [22, 23]: White Esthetic Score (WES) and Pink Esthetic Score (PES). WES is an aesthetic evaluation system employed to assess the beauty of anterior dental elements, including implant crowns. It considers various factors such as shape, size, position, coloration, and texture of the dental crown. Its purpose is to provide an objective assessment of the aesthetic appearance, enabling dental professionals to evaluate the aesthetic quality of anterior dental restorations. PES is a specifically designed evaluation system to assess the aesthetics of the gingival area, particularly around dental implants. It considers parameters such as gum color, shape and size of the gingival tissue, presence of gingival recessions, and the harmonious transition between the implant restoration and the surrounding tissue. The goal of PES is to provide a comprehensive assessment of the aesthetics of the gingiva around dental implants, thereby contributing to achieving optimal aesthetic results in the gingival area. Both systems, WES and PES, are valuable tools for evaluating the overall aesthetics of implant restorations. The combined use of these approaches allows for a comprehensive assessment that takes into consideration both the appearance of dental crowns and the health and aesthetics of the gingiva surrounding the implants [22, 23].

In conclusion, the evaluation of OHRQoL is essential in implant rehabilitation as it provides a comprehensive understanding of the treatment’s impact on the patient’s life, contributing to delivering more personalized, effective, and well-being-oriented care.

To date, several studies have assessed patient satisfaction and OHRQoL regarding implant-prosthetic rehabilitation such as impact on quality of life in overdentures (a type of removable denture resting on the remaining natural teeth, teeth root, or dental implants) retained by mini-dental implants (MDIs), the evaluation of patient experiences with implant treatments performed under general anesthesia, the assessment of tissue stability and aesthetic perception in single immediate implants in the esthetic zone, the analysis of variations in dental anxiety, aesthetic perception, and OHRQoL after anterior implant treatment, the evaluation of patient satisfaction and prosthetic complications of different types of maxillary and mandibular prostheses, the comparison between fixed prostheses supported by zygomatic implants and all-on-four prostheses, the investigation of changes in phonetics, satisfaction, and quality of life in patients with maxillary overdentures, the comparison of satisfaction and quality of life among different types of prostheses, patient-reported outcome measures of soft tissue substitutes versus autogenous grafts for soft tissue augmentation procedures, and the comparison between fixed and overdenture prostheses supported by zygomatic implants [24–31]. However, few studies to date have compared changes in OHRQoL from temporary implant-supported prostheses to permanent implant-supported prostheses, whether crowns, bridges, overdentures, and Toronto-type prostheses i.e., complete fixed prostheses with a flange, replacing up to 12 teeth per arch, fixed by abutments on dental implants using the immediate-load implant technique or, in more traditional dentistry, with deferred-load implant dentistry [32–37].

Therefore, the primary aim of this prospective clinical study was to assess OHRQoL perceived by the patient through OHIP-14 questionnaire before (baseline), during (provisional prostheses), and after (definitive prostheses) implant-prosthetic rehabilitation, also considering patients’ and interventions’ characteristics.

The secondary aims were to assess patients’ perceived aesthetics through VAS scale before (baseline), during (provisional prosthesis), and after (definitive prosthesis) implant-prosthetic rehabilitation; to explore the impact of interventions on specific domains of OHIP-14, as measured by changes in scores across seven domains.

## Methods

### Study design

This study was approved by the Ethics Committee of the Fondazione IRCCS Cà Granda Ospedale Maggiore Policlinico, No. 864_2021 (Trial ID 2444) and was held according to the Helsinki statements. The study follows the STrengthening the Reporting of OBservational studies in Epidemiology (STROBE) guidelines shown in Table S1 (Supplementary Materials) [38]. A prospective single-center observational cohort study was conducted with patients who required implant-prosthetic rehabilitation, recruited from the Implant Center for Edentulism and Jawbone Atrophies, Maxillo-Facial Surgery and Dental Unit of the Fondazione IRCCS Cà Granda Ospedale Maggiore Policlinico (Italy), and performed by the same oral surgeon and prosthodontist, both with more than 15 years of experience. The volunteers were recruited between September 2021 and June 2022. All recruited subjects were informed about the objectives and study design, and those who consented to participate signed a written informed consent form.

### Study population

The study population consisted of all fully or partially edentulous patients who presented for a visit to the Implant Center for Edentulism and Jawbone Atrophies requesting implant-prosthetic rehabilitation, and who met the eligibility criteria adopted at the same department in compliance with current clinical practice to be able to place and rehabilitate dental implants safely and predictably.

### Eligibility criteria

In this prospective study, all patients who received implant-prosthetic rehabilitation were consecutively enrolled. Criteria for the selection of candidate patients to receive implants generally included the following: male or female patients, partially toothed or edentulous, aged ≥ 18 years, in good general health through the American Society of Anesthesiologists (ASA) scale, i.e., ASA I or II, in need of implant-prosthetic rehabilitation in the anterior and/or posterior sectors of the maxillary upper jaw and/or mandible, with adequate oral hygiene (Simplified Oral Hygiene Index (OHI-S) score [39] ≤ 1.2 and Modified Sulcus Bleeding Index (mSBI) [40] score 0), able to understand the nature of the proposed questionnaire fully, and able to sign the informed consent form. Additional exclusion criteria-local, systemic, and related to the patient’s habits and lifestyle-were adopted on a case-by-case basis according to current clinical practice.

### Endpoints and survey description

For the assessment of the patient’s perceived OHRQoL before (baseline–TB), during (provisional prostheses–TP), and after treatment (definitive prostheses –TD) by implant-prosthetic rehabilitations, a single questionnaire was used, given to the patient at TB, TP, and TD. The questionnaire was based on the OHIP-14, which consisted of 14 questions divided into 7 domain items: functional limitation, physical pain, psychological discomfort, physical disability, psychological disability, social disability, and handicap [16]. Table S2 (Supplementary Material) shows the OHIP-14 questionnaire.

In the present study, the Italian version, IOHIP-14 was used, which had been validated and had good equivalence to its original OHIP-14 version [41]. For each of the 14 questions corresponding to the 7 domains related to a particular aspect of perceived oral health status, the subject responded by choosing the most appropriate one from among 5 response levels, with a score between 1 and 5 (1 = never; 2 = hardly never; 3 = occasionally; 4 = fairly often; 5 = very often). Hence, a domain score ranges from 2 to 10 points: scores 2–4, minimal impact; scores 5–7, moderate impact; scores 8–10, high impact. The OHIP-14 scores, ranging from 14 to 70, were computed by summing the ordinal values assigned to the 14 items, where higher OHIP-14 scores signified poorer (43–56, significant impact; 57–70, high impact) and lower scores signified improved OHRQoL (14–28, minimal impact; 29–42, moderate impact) [16].

Patients’ perceived aesthetic was analyzed through a VAS scale from 0 to 100: scores 0–20 (very low score) - poor aesthetic perception or experience; scores 21–40 (low score) - negative aesthetic perception or experience; scores 41–60 (medium score) - moderately negative or neutral aesthetic perception or experience; scores 61–80 (high score) - positive aesthetic perception or experience; scores 81–100 (very high score) - excellent aesthetic perception or experience [42].

For the primary endpoint, OHRQoL summary score obtained by each patient on the 14 questions of the OHIP-14 questionnaire at TB, TP, and TD was calculated.

For the secondary endpoints, patients’ perceived aesthetics through VAS score obtained by each patient at TB, TP, and TD was evaluated; OHRQoL summary score and patients’ aesthetic perception by VAS score were compared with biological sex, number of substitutions, and intervention in the aesthetic areas (between second upper premolars, 1.5 and 2.5) [43]; potential changes in the seven domains of OHIP-14 from TB, to TP and TD were analyzed.

There were no follow-up visits after TD. The study duration was variable according to the patient, being related to the duration of the patient’s planned treatment.

### Planned visits and operating protocol

Each patient was enrolled in the present protocol after anamnestic framing and acceptance of the treatment plan. For the conduct of the present study, three visits were required for the delivery/collection of questionnaires: an initial visit (TB), a follow-up visit 3 months after completion of provisional implant-prosthetic rehabilitation (TP), and a follow-up visit 3 months after completion of definitive implant-prosthetic rehabilitation (TD).

During the first visit (TB), as per practice, the medical history was collected, and a clinical and radiographic evaluation was performed to verify the patient’s eligibility for implant-prosthetic rehabilitation according to the eligibility criteria. Patients who met these requirements and agreed to be rehabilitated according to the planned treatment plan were offered to participate in the present study for OHRQoL evaluation explaining the rationale and operative protocol. Patients who agreed to participate in the present study were given the questionnaire for evaluation of OHRQoL at TB, and the rest of the protocol forms. The questionnaire and protocol forms, completed by the patient, were collected at the next visit.

Each patient followed the accepted implant-prosthetic treatment pathway discussed during the formulation of the treatment plan according to current clinical practice, with no difference from the treatment pathway that may have been proposed if the patient’s consent to be enrolled in this protocol was denied.

Regarding surgical procedures, when a three-dimensional bone is sufficient to allow guided insertion of one or more implants, our approach has focused on preparing full-thickness flaps with submerged healing of the implant for a period ranging from 3 to 6 months, depending on the arch involved (mandible or maxilla). In cases where bone regeneration is deemed necessary, our practice involves the adoption of personalized guided bone regeneration (GBR) techniques, ensuring a targeted approach to the specifics of each case. The distinguishing feature of our approach lies in the guided prosthetic placement of implants, a crucial criterion for assessing the need for bone regeneration procedures.

Regarding the prosthetic phase, the management of single crowns and implant-supported bridges involved separate two-stage procedures. It is noteworthy that type-Toronto prostheses can be loaded immediately, provided primary implant stability is achieved at a minimum of 35 N/cm. Alternatively, a staggered approach with submerged healing is used, with an implementation period ranging from 3 to 6 months depending on the dental arch. During this phase, management of removable complete dentures is critical to distribute the forces on the implants adequately.

After completing their provisional and definitive implant-prosthetic rehabilitations at 3 months (TP and TD, respectively), patients were given the same questionnaire they received initially (TB) to evaluate their perceived oral health-related quality of life (OHRQoL). They were asked to return the completed questionnaire within 7 days on both occasions.

All implant-prosthetic rehabilitations were performed by the same oral surgeon and prosthetist, both with more than 15 years of experience. Two other different oral surgeons from the same group of dentists previously reported performed the collection of research data.

### Data collection

Data on patients included in the study were collected in dedicated data collection forms. The source documents were outpatient medical records in which all data on the treatment plan, interventions, prosthetic steps, and follow-up visits were recorded. The data acquired from the questionnaires and medical records needed for the study were transferred and recorded in an electronic database (Excel, Microsoft Corporation).

### Sample size calculation

We employed dedicated software (PASS Sample Size Software, NCSS LCC) for sample size calculation. The sample size was calculated from pilot data obtained from 20 questionnaires (20 patients). The results of these 20 patients were not included in the protocol. Assuming a positive response to perceived quality of life, as indicated by an OHIP-14 questionnaire score ranging from 14 to 25, and considering our previous conversation, we established that 40% of participants would exhibit positive responses during baseline assessment (TB). We anticipated this proportion to rise to 60% during definitive prostheses assessment (TD) for the same individuals. To maintain a first-type error rate of 5% and achieve an 80% power in detecting a difference in positive response rates between TB and TD, we determined a minimum sample size of 100 patients for our study.

### Statistical methods

Interval scale variables were summarized as median and interquartile range (IQR). Categorical variables were summarized as absolute and relative frequencies. Both patient- and prosthesis-level variables were collected. Since the outcome variables of interest OHRQoL and VAS are patient-level variables, the analyses were carried out using patient-levels variables, namely biological sex, age, intervention(s) considered to be in an aesthetic area, and the dichotomized number of implants (up to 3 versus more than 3). Prosthesis-level variables are used for descriptive purposes only (i.e., for describing the sample). In the analyses, 1 patient with only Toronto-type prostheses was excluded because it is uncommon while retaining patients with Toronto-type prostheses and simultaneously crowns/bridges on implants for a patient to have only Toronto-type prostheses because the responses are by patient. To initially explore the data, the non-parametric Friedman test was employed to identify any overall trends or significant differences across interventions (TB to TD) and subgroups (e.g., males and females). The Friedman test was applied both to the final OHRQoL and VAS scores and the sub-domains in the questionnaire. For the total OHRQoL and VAS scores, the Friedman tests served as preliminary insight before a Generalized Linear Mixed Effect Model (GLMM) and Linear Mixed Effect Model (LMM) were employed for studying the total OHRQoL and VAS scores, respectively. While, for the domains, the Friedman tests were employed, with the Bonferroni correction for multiple comparisons, as exploratory analysis for future work.

For the total OHRQoL score variable only values up to 42 were recorded. Therefore, it was dichotomized using the groups indicated in Sect. 2.4, namely minimal impact versus moderate impact. A random intercept only, the patient, GLMM was employed to investigate the effect of intervention stages, demographic characteristics, and procedures variables on the total OHRQoL score. Specifically, other than the intervention stage, biological sex, dichotomized age (patients younger than 65 years were considered *young*, patients 65 years old or older were considered *old*), the general area of the interventions (aesthetic versus non-aesthetic), and the dichotomized number of substitutions (up to 3 versus more than 3) variables were considered. Alternative models, i.e., with a lower number of covariates (but never discarding the intervention/time variable) were considered and compared by mean of the Akaike Information Criteria (AIC). However, since there was no significant improvement in the AIC, the complete model was retained. Analogously, an intercept only LMM was used to study the effects of the same variables on the VAS score.

All the analyses were obtained using R [44], version 4.1.2 (2021-11-01). The GLMM and LMM models estimates were obtained through *lme4* package (version 3.1.3) [45].

## Results

Table 1 reports a summary of the characteristics of patients and implant. A total of 99 patients (35 males and 64 females, median age 67 (61–74) years) rehabilitated with 26 single crowns, 127 implant-supported bridges, and 2 Toronto-type prostheses were enrolled. Regarding number of implants, 29 single crowns and 116 implant-supported bridges were performed with ≤ 3 implants; instead, 13 implant-supported bridges and 2 Toronto-type prostheses were performed with > 3 implants. Patients’ responses are summarized in Table 2.

**Table 1:** Patients’ and prostheses characteristics

Patient-level and Implant-level characteristics (*n* = 99 and 416, respectively)		
Age (years), median [IQR]		67 [61, 74]
Biological sex, n. (%)	Female	64 (64.6)
	Male	35 (35.4)
Aesthetic area, n. (%)	No	16 (16.2)
	Yes	83 (83.8)
Number of sostitutions, n. (%)	More than 4	45 (45.5)
	Up to 3	54 (54.5)
Prostheses type, n. (%)	Crown	27 (65.0)
	Bridge	379 (91.1)
	Toronto	10 (2.4)

IQR, interquartile range

**Table 2:** Patients’ responses to outcomes analyzed at TB, TP, and TD.

	TB	TP	TD
Aesthetic area			
Mean (SD)	1.2 (0.37)	1.2 (0.37)	1.2 (0.37)
**Functional limitation**			
Mean (SD)	3.6 (2.1)	3.2 (1.5)	2.7 (1.2)
**Physical pain**			
Mean (SD)	4.0 (2.2)	3.2 (1.6)	2.8 (1.4)
**Psychological discomfort**			
Mean (SD)	4.1 (2.3)	3.0 (1.3)	2.7 (1.1)
**Physical disability**			
Mean (SD)	4.1 (2.2)	3.4 (1.8)	2.8 (1.5)
**Psychological disability**			
Mean (SD)	3.9 (2.1)	3.2 (1.7)	2.7 (1.2)
**Social disability**			
Mean (SD)	3.8 (2.2)	3.3 (1.9)	2.6 (1.1)
**Handicap**			
Mean (SD)	4.1 (2.4)	3.1 (1.8)	2.7 (1.2)
**OHRQoL summary score**			
Mean (SD)	28 (7.6)	22 (5.5)	19 (4.7)
**VAS**			
Mean (SD)	29 (22)	59 (18)	81 (14)

OHRQoL, oral health-related quality of life; SD, standard deviation; VAS, visual analogue scale; TB, baseline; TD, definitive prostheses; TP, provisional prostheses

### OHRQoL summary score

Table 3 shows the parameters estimated from the GLMM model for the OHRQoL summary score. The OHRQoL summary score is significantly affected by the intervention steps. Compared to the baseline, the odds of a worse quality of life, specifically from minimal to moderate impact of oral conditions on patients’ well-being, after the interventions for the provisional and definitive prosthesis/es were significantly lower, with odds ratios of 0.04 (95% CI: 0.01–0.18, p-value < 0.001) and 0.01 (CI: 0.00–0.05, p-value < 0.001), respectively. Other predictors, such as biological sex, with an odds ratio of 2.32 (CI: 0.45–11.88, p-value = 0.312), the number of substitutions with an odds ratio of 0.89 (CI: 0.18–4.31, p-value = 0.880), and intervention(s) in an aesthetic area did not reach show statistical significance.

**Table 3:** Random-intercept generalized regression model for dichotomized OHRQoL summary score. The subject-specific regression coefficients are reported and equipped with standard error, 95% confidence interval (95% CI) and p-value

	OHRQoL (minimal vs. moderate impact)		
*Predictors*	*Odds Ratios*	*95% CI*	*p-value*
(Intercept)	0.65	0.06–6.90	0.722
Provisional prosthesis/es vs. Baseline	0.04	0.01–0.18	**< 0.001**
Definitive prosthesis/es vs. Baseline	0.01	0.00–0.05	**< 0.001**
Biological sex [Male vs. Female]	2.32	0.45–11.88	0.312
N. of substitutions [more than 4 vs. up to 3]	0.89	0.18–4.31	0.880
Aesthetic area [Yes vs. No]	0.79	0.10–6.51	0.825
**Random Effects**			
σ^2^	3.29		
σ^2^_Patient_	9.10		

CI, confidence interval; OHRQoL, oral health-related quality of life

### VAS score

Table 4 reports the parameters estimated from the LMM model for the VAS score. The model shows that the use of provisional prostheses led to a significant increase in VAS scores compared to baseline, with an estimated increase of 30.44 points (CI: 26.60–34.29, p-value < 0.001). Similarly, the use of definitive prostheses was associated with an increase estimated at 51.97 points (CI: 48.12–55.81, p-value < 0.001) compared to baseline. Other predictors, such as biological sex and the number of substitutions, as well as the intervention(s) in aesthetic areas, did not show statistically significant effects on the VAS score. The estimated effect for biological sex was 4.83 points (CI: -1.13–10.79, *p* = 0.112), for the number of substitutions was 0.88 points (CI: -4.93–6.69, *p* = 0.766), and for the aesthetic area was 1.17 points (CI: -6.63–8.97, *p* = 0.768). The random effects in the model, which account for individual variability among patients, were also significant. The patient-level variability was estimated at 140.89 (42.72%), contributing to an Intraclass Correlation Coefficient (ICC) of 0.43. This indicates that 43% of the total variability in VAS scores can be attributed to differences between patients.

**Table 4:** Random intercept linear regression model for VAS score

	VAS		
*Predictors*	*Estimates*	*CI*	*p-value*
(Intercept)	25.55	16.67–34.42	**< 0.001**
Provisional prosthesis/es vs. Baseline	30.44	26.60–34.29	**< 0.001**
Definitive prosthesis/es vs. Baseline	51.97	48.12–55.81	**< 0.001**
Biological sex [Male vs. Female]	4.83	-1.13–10.79	0.112
N. of substitutions [more than 4 vs. up to 3]	0.88	-4.93–6.69	0.766
Aesthetic area [Yes vs. No]	1.17	-6.63–8.97	0.768
**Random Effects**			
σ^2^	188.90		
σ^2^_Patient_	140.89		
ICC	0.43		

CI, confidence interval; ICC, intraclass correlation coefficient; VAS, visual analogue scale

### OHIP-14 domains

Preliminary results on the variation of the domain-specific scores between intervention steps suggested that significant changes occurred in each of the seven domains when comparing the scores obtained at baseline, after the provisional prosthesis intervention, and after the definitive prosthesis intervention. The consistent significance across all domains indicates a systematic influence of the interventions on the domain-specific outcomes, with adjustments in prosthesis leading to measurable improvements or changes in each evaluated aspect. This observation serves as a preliminary result, highlighting areas for further in-depth exploration in future studies to better understand the specific impacts and implications of each intervention step on the domain-specific outcomes.

## Discussion

The present study aimed to evaluate the change in OHRQoL and aesthetic smile satisfaction using the VAS scale before, during, and after implant-prosthetic rehabilitation for missing teeth and to analyze the physical and psychological impact of dental implants and related prosthetic restorations. Potential variables influencing total OHRQoL, derived from the sum of the 7 domains, were considered.

Regarding the OHRQoL summary score, the implementation of provisional and definitive prostheses significantly reduced the odds of worsening patients’ quality of life, especially their general well-being compared with oral conditions. This is evidenced by the low values of odds ratios for both types of prostheses, indicating a moderate improvement in the oral health-related quality of life of patients. Additionally, it should be noted that, for the considered sample, there was a transition from moderate impact to minimal impact concerning OHRQoL. This had been partially confirmed by Winter et al. [46], who showed significant improvements in OHRQoL only with definitive prostheses.

However, other factors such as biological sex and number of replacements did not show statistical significance in the analysis. Interestingly, males had higher OHRQoL scores, suggesting a greater perception of the impact of oral health on quality of life than females, in contrast to a recent prospective study by Nickenig et al. [47], which showed equal OHRQoL scores between males and females. In addition, patients with more than 4 dental implants have higher mean OHRQoL scores, indicating a greater impact of dental implants on their quality of life, in contrast to the clinical trial by Passia et al. [48], which showed that OHRQoL increased regardless of the number of implants.

Regarding variation in OHIP-14 domains, preliminary results indicate significant changes in different domains of oral health-related quality of life after intervention with provisional and definitive dentures. This suggests that such interventions have a systemic influence on different aspects of patient’s well-being, with measurable improvements in each domain assessed.

VAS scores provide a significant increase with both provisional and definitive prostheses compared with the baseline value, concerning patients’ perceived aesthetics. This shows a subjective improvement in patients’ perceived aesthetic well-being after prosthetic surgery, with an estimated increase of 30.44 points for provisional prostheses and 51.97 points for definitive prostheses, in contrast with the consensus report of Feine et al. [49], who showed that the use of a provisional restoration did not affect patients’ evaluation of the aesthetics of permanent restorations on implant-supported FDPs.

However, other factors such as biological sex and number of substitutions did not show a significant impact on VAS scores. Although males had higher VAS scores on average this difference was not statistically significant, in agreement with the study by Wang et al. [50]. Also, the number of substitutions did not seem to influence VAS scores significantly.

Interventions in aesthetic areas appear to lead to a greater increase in VAS scores. Although this difference was not statistically significant, it might suggest that patients give more importance to the aesthetic aspects of prostheses, according to Baracat et al. [51].

Finally, the results also show significant individual variability among patients, with 43% of the total variation in VAS scores attributed to differences between patients. This underscores the importance of considering individual patient characteristics when interpreting results and planning treatment.

Future studies can be conducted to define the impact of selected restorative materials in implant-prosthetic rehabilitation (crowns, bridges, Toronto prostheses) on patient perception and their OHRQoL. Subsequent research endeavors could delve deeper into assessing the OHRQoL following implant-prosthetic rehabilitation in individuals with disabilities [52]. Such studies could explore the efficacy of different rehabilitation approaches, the impact of regular follow-up on OHRQoL outcomes, and the effectiveness of training programs in enhancing communication and care for this unique patient demographic. Finally, considering the growing emphasis on objective aesthetic evaluation criteria in dental research, it becomes imperative to advocate for future studies that delve deeper into the nuances of esthetic outcomes in implant dentistry. The existing literature provides a glimpse into the promising realm of single-tooth implant procedures in the anterior region, particularly those employing a flapless approach and custom-made zirconia-ceramic components [53].

Several limitations require consideration in the interpretation of the findings. This is a single-center study without a control group and a small sample size related to the number of variables: different types of prostheses and dental arch could have different results when analyzed together (multivariable model), particularly for mandibular full-arch prostheses. In addition, the number of prosthetic-Toronto-type rehabilitations performed is limited compared to prosthetic rehabilitations with single crown and implant-supported bridges. In addition, patients might have remembered the answers given to the OHIP-14 questionnaire and the VAS scale considering that they were applied three times in a short period. Another limitation is the short-term evaluation of the OHIP-14 (less than one year in total), which might differ from the patient’s perception after several years of dentures regarding OHRQoL, function, and any problems. The recruitment of partially or fully edentulous patients could be an influencing factor in the perceived patients’ responses. We used the 7-domain OHIP-14 questionnaire instead of the new concept of 4 dimensions of OHIP considering the need to use the validated questionnaire in the Italian language; finally, patients’ aesthetic perception was not assessed by PES and WES scores but only by VAS scale.

## Conclusion

In conclusion, it can be said that implant-prosthetic rehabilitations lead to significant improvement in OHRQoL and smile aesthetic satisfaction in edentulous or partially edentulous patients. In general, regardless of the variables analyzed, reported substantial improvement in OHRQoL at both provisional and final prosthetic delivery, with significant differences from baseline. Thus, the provisional stage becomes critical not only to restore proper stomatognathic function but also to guide the healing of the peri-implant soft tissues to achieve an ideal architecture and anatomy at the time of delivery of the final prosthesis.

The change in OHRQoL is accompanied by a marked improvement in the patient’s aesthetic perception of the new smile similar in all intervals of the study. Finally, the OHRQoL could provide the basis for any dental health care program and should be considered an important element in the overall oral health program because it allows the focus to shift not only to clinical-radiographic variables but also to more subjective elements related to the patient himself to improve current clinical practice toward patients.

### Electronic supplementary material

Below is the link to the electronic supplementary material.

Supplementary Material 1

## Data Availability

The data are available for use upon request to the corresponding author.

## Abbreviations

ASA: American Society of Anesthesiologists
HRQoL: Health-Related Quality of Life
IOHIP-14: Italian Oral Health Impact Profile-14
IQR: Interquartile Range
mSBI: Modified Sulcus Bleeding Index
OHI-S: Simplified Oral Hygiene Index
OHIP-14: Oral Health Impact Profile-14
OHIP-49: Oral Health Impact Profile-49
OHIP: Oral Health Impact Profile
OHRQoL: Oral Health-Related Quality of Life
PES: Pink Esthetic Score
SD: Standard Deviation
STROBE: Strengthening the Reporting of Observational studies in Epidemiology
VAS: Visual Analogue Scale
WES: White Esthetic Score
WHO: World Health Organization

## References

[1] Awad MA, Lund JP, Shapiro SH, Locker D, Klemetti E, Chehade A, Savard A, Feine JS (2003). Oral health status and treatment satisfaction with mandibular implant overdentures and conventional dentures: a randomized clinical trial in a senior population. Int J Prosthodont.

[2] Slade GD (2012). Oral health-related quality of life is important for patients, but what about populations?. Commun Dent Oral Epidemiol.

[3] WHO. (1948). World Health Organization Constitution. Geneva, Switzerland: World Health Organization; Retrieved January 18, 2011, from http://www.who.int/governance/eb/who_constitution_en.pdf.

[4] Gift HC, Atchison KA, Dayton CM (1997). Conceptualizing oral health and oral health-related quality of life. Soc Sci Med.

[5] Davis P (1976). Compliance structures and the delivery of health care: the case of dentistry. Soc Sci Med.

[6] Cohen LK, Jag JD (1976). Toward the formulation of sociodental indicators. Int J Health Serv.

[7] Bennadi D, Reddy CVK (2013). Oral health related quality of life. J Int Soc Prev Community Dent.

[8] Cushing AM, Sheiham A, Maizels J (1986). Developing socio-dental indicators–the social impact of dental disease. Community Dent Health.

[9] Ettinger RL (1987). Oral disease and its effect on the quality of life. Gerodontics.

[10] Al Shamrany M (2006). Oral health-related quality of life: a broader perspective. East Mediterr Health J.

[11] Rockville MD. Mental health: A report of the Surgeon General. US Department of Health and Human Services 1999. https://profiles.nlm.nih.gov/101584932X120.

[12] Alvarez-Azaustre MP, Greco R, Llena C (2021). Oral health-related quality of life in adolescents as measured with the Child-OIDP questionnaire: a systematic review. Int J Environ Res Public Health.

[13] Locker D, Miller Y (1994). Evaluation of subjective oral health status indicators. J Public Health Dent Summer.

[14] Petersen PE (2003). The world oral health report 2003: continuous improvement of oral health in the 21st century–the approach of the WHO Global Oral Health Programme. Community Dent Oral Epidemiol.

[15] Slade GD, Spencer AJ (1994). Development and evaluation of the oral Health Impact Profile. Community Dent Health.

[16] Slade GD (1997). Derivation and validation of a short-form oral health impact profile. Community Dent Oral Epidemiol.

[17] Gonçalves GSY, de Magalhães KMF, Rocha EP, Dos Santos PH, Assunção WG (2022). Oral health-related quality of life and satisfaction in edentulous patients rehabilitated with implant-supported full dentures all-on-four concept: a systematic review. Clin Oral Investig.

[18] Nickenig HJ, Terheyden H, Reich RH, Kreppel M, Linz C, Lentzen MP (2023). Oral health-related quality of life (OHRQoL) and implant therapy: a prospective multicenter study of preoperative, intermediate, and posttreatment assessment. J Craniomaxillofac Surg.

[19] Papaspyridakos P, Chen CJ, Singh M, Weber HP, Gallucci GO (2012). Success criteria in implant dentistry: a systematic review. J Dent Res.

[20] Cosyn J, Thoma DS, Hämmerle CH, De Bruyn H (2017). Esthetic assessments in implant dentistry: objective and subjective criteria for clinicians and patients. Periodontol 2000.

[21] Buser D, Mericske-Stern R, Bernard JP, Behneke A, Behneke N, Hirt HP, Belser UC, Lang NP (1997). Long-term evaluation of non-submerged ITI implants. Part 1: 8-year life table analysis of a prospective multi-center study with 2359 implants. Clin Oral Implants Res.

[22] Sanchez-Perez A, Nicolas-Silvente AI, Sanchez-Matas C, Molina-García S, Navarro-Cuellar C, Romanos GE (2021). Primary stability and PES/WES evaluation for immediate implants in the aesthetic zone: a pilot clinical double-blind randomized study. Sci Rep.

[23] Foong ALY, Tey VHS, Tan KBC, Teoh KH, Tan K (2022). Esthetic evaluation of Anterior Implant-supported single crowns: a comparison between patients and dentists. Int J Prosthodont.

[24] Van Doorne L, Fonteyne E, Matthys C, Bronkhorst E, Meijer G, De Bruyn H (2021). Longitudinal oral health-related quality of life in maxillary mini dental implant overdentures after 3 years in function. Clin Oral Implants Res.

[25] Matthys C, Vervaeke S, Besseler J, De Bruyn H (2019). Five-year study of mandibular overdentures on stud abutments: clinical outcome, patient satisfaction and prosthetic maintenance-influence of bone resorption and implant position. Clin Oral Implants Res.

[26] Gjelvold B, Kisch J, Chrcanovic BR, Albrektsson T, Wennerberg A (2017). Clinical and radiographic outcome following immediate loading and delayed loading of single-tooth implants: randomized clinical trial. Clin Implant Dent Relat Res.

[27] Thoma DS, Strauss FJ, Mancini L, Gasser TJW, Jung RE (2023). Minimal invasiveness in soft tissue augmentation at dental implants: a systematic review and meta-analysis of patient-reported outcome measures. Periodontol 2000.

[28] Sidenö L, Hmaidouch R, Brandt J, von Krockow N, Weigl P (2018). Satisfaction level in dental-phobic patients with implant-supported rehabilitation performed under general anaesthesia: a prospective study. BMC Oral Health.

[29] Xie X, Zhang Z, Zhou J, Deng F (2023). Changes of dental anxiety, aesthetic perception and oral health-related quality of life related to influencing factors of patients’ demographics after anterior implant treatment: a prospective study. Int J Implant Dent.

[30] Fayek NH, Mahrous AI, Shaaban AAE, ELsyad MA (2022). Patient satisfaction and prosthetic complications of Maxillary Implant overdentures Opposing Mandibular Implant overdentures with Bar, Telescopic, and Stud attachments: a 1-Year prospective trial. Int J Oral Maxillofac Implants.

[31] Fernández-Ruiz JA, Sánchez-Siles M, Guerrero-Sánchez Y, Pato-Mourelo J, Camacho-Alonso F (2021). Evaluation of quality of life and satisfaction in patients with fixed prostheses on zygomatic implants compared with the All-on-four Concept: a prospective Randomized Clinical Study. Int J Environ Res Public Health.

[32] Raes F, Cosyn J, De Bruyn H (2013). Clinical, aesthetic, and patient-related outcome of immediately loaded single implants in the anterior maxilla: a prospective study in extraction sockets, healed ridges, and grafted sites. Clin Implant Dent Relat Res.

[33] Raes S, Raes F, Cooper L, Giner Tarrida L, Vervaeke S, Cosyn J, De Bruyn H (2017). Oral health-related quality of life changes after placement of immediately loaded single implants in healed alveolar ridges or extraction sockets: a 5-year prospective follow-up study. Clin Oral Implants Res.

[34] Barroso-Panella A, Ortiz-Puigpelat O, Altuna-Fistolera P, Lucas-Taulé E, Hernández-Alfaro F, Gargallo-Albiol J (2020). Evaluation of peri-implant tissue Stability and patient satisfaction after Immediate Implant Placement in the esthetic area: a 3-Year follow-up of an ongoing prospective study. Int J Periodontics Restor Dent.

[35] Fonteyne E, Van Doorne L, Becue L, Matthys C, Bronckhorst E, De Bruyn H (2019). Speech evaluation during maxillary mini-dental implant overdenture treatment: a prospective study. J Oral Rehabil.

[36] Sánchez-Torres A, Moragón-Rodríguez M, Agirre-Vitores A, Cercadillo-Ibarguren I, Figueiredo R, Valmaseda-Castellón E. Early complications and quality of life in patients with immediately loaded implant-supported maxillary partial rehabilitations: a prospective cohort study. Med Oral Patol Oral Cir Bucal. 2023;26158. 10.4317/medoral.26158. 10.4317/medoral.26158 PMC10945874 37471302

[37] Van Doorne L, Vandeweghe S, Matthys C, Vermeersch H, Bronkhorst E, Meijer G, De Bruyn H (2023). Five years clinical outcome of maxillary mini dental implant overdenture treatment: a prospective multicenter clinical cohort study. Clin Implant Dent Relat Res.

[38] von Elm E, Altman DG, Egger M, Pocock SJ, Gøtzsche PC, Vandenbroucke JP, STROBE Initiative (2008). The strengthening the reporting of Observational studies in Epidemiology (STROBE) statement: guidelines for reporting observational studies. J Clin Epidemiol.

[39] Greene JC, Vermillion JR (1964). The simplified oral Hygiene Index. J Am Dent Assoc.

[40] Pontoriero R, Tonelli MP, Carnevale G, Mombelli A, Nyman SR, Lang NP (1994). Experimentally induced peri-implant mucositis. A clinical study in humans. Clin Oral Implants Res.

[41] Corridore D, Campus G, Guerra F, Ripari F, Sale S, Ottolenghi L (2014). Validation of the Italian version of the oral Health Impact Profile-14 (IOHIP-14). Ann Stomatol (Rome).

[42] Burgueño-Barris G, Cortés-Acha B, Figueiredo R, Valmaseda-Castellón E (2016). Aesthetic perception of single implants placed in the anterior zone. A cross-sectional study. Med Oral Patol Oral Cir Bucal.

[43] Testori T, Weinstein T, Scutellà F, Wang HL, Zucchelli G (2018). Implant placement in the esthetic area: criteria for positioning single and multiple implants. Periodontol 2000.

[44] R Core Team. (2021). R: A language and environment for statistical computing. R Foundation for Statistical Computing, Vienna, Austria. https://www.R-project.org/.

[45] Bates D, Maechler M, Bolker B, Walker S (2015). Fitting Linear mixed-effects models using lme4. J Stat Softw.

[46] Winter A, Erdelt K, Giannakopoulos NN, Schmitter M, Edelhoff D, Liebermann A (2021). Impact of different types of dental prostheses on oral-health-related quality of life: a prospective bicenter study of definitive and interim restorations. Int J Prosthodont.

[47] Nickenig HJ, Terheyden H, Reich RH, Kreppel M, Linz C, Lentzen MP (2024). Oral health-related quality of life (OHRQoL) and implant therapy: a prospective multicenter study of preoperative, intermediate, and posttreatment assessment. J Craniomaxillofac Surg.

[48] Passia N, Chaar MS, Krummel A, Nagy A, Freitag-Wolf S, Ali S, Kern M (2022). Influence of the number of implants in the edentulous mandible on chewing efficacy and oral health-related quality of life-A within-subject design study. Clin Oral Implants Res.

[49] Feine J, Abou-Ayash S, Al Mardini M, de Santana RB, Bjelke-Holtermann T, Bornstein MM, Braegger U, Cao O, Cordaro L, Eycken D, Fillion M, Gebran G, Huynh-Ba G, Joda T, Levine R, Mattheos N, Oates TW, Abd-Ul-Salam H, Santosa R, Shahdad S, Storelli S, Sykaras N, Treviño Santos A, Stephanie Webersberger U, Williams MAH, Wilson TG, Wismeijer D, Wittneben JG, Yao CJ, Zubiria JPV (2018). Group 3 ITI Consensus Report: patient-reported outcome measures associated with implant dentistry. Clin Oral Implants Res.

[50] Wang Y, Bäumer D, Ozga AK, Körner AG, Bäumer A (2021). Patient satisfaction and oral health-related quality of life 10 years after implant placement. BMC Oral Health.

[51] Baracat LF, Teixeira AM, dos Santos MBF, de Cunha P, Marchini V. L. Patients’ expectations before and evaluation after dental implant therapy. Clin Implant Dent Relat Res. 2011;13:141–145. 10.1111/j.1708-8208.2009.00191.x. 10.1111/j.1708-8208.2009.00191.x 19681923

[52] D’Addazio G, Santilli M, Sinjari B, Xhajanka E, Rexhepi I, Mangifesta R, Caputi S (2021). Access to Dental Care-A Survey from dentists, people with disabilities and caregivers. Int J Environ Res Public Health.

[53] Traini T, Pettinicchio M, Murmura G, Varvara G, Di Lullo N, Sinjari B, Caputi S. Esthetic outcome of an immediately placed maxillary anterior single-tooth implant restored with a custom-made zirconia-ceramic abutment and crown: a staged treatment. Quintessence Int;42:103–8. 21359243

