# Supplementary material for: Oral health-related quality of life in implant-supported rehabilitations: a prospective single-center observational cohort study
Source: BMC Oral Health. 2024 May 4;24:531. doi: 10.1186/s12903-024-04265-y (PMC11069144; doi:10.1186/s12903-024-04265-y)
Supplement: Supplementary file 1 — Supplementary Material 1 [file 12903_2024_4265_MOESM1_ESM.docx]

**Table S1.** STROBE Checklist of cohort studies.

|  | Item No | Recommendation | Reported On Page # |
| --- | --- | --- | --- |
| **Title and abstract** | 1 | (*a*) Indicate the study’s design with a commonly used term in the title or the abstract | Title |
|  |  | (*b*) Provide in the abstract an informative and balanced summary of what was done and what was found | Abstract |
| Introduction | | |  |
| Background/rationale | 2 | Explain the scientific background and rationale for the investigation being reported | Initial introduction |
| Objectives | 3 | State specific objectives, including any prespecified hypotheses | End of introduction |
| Methods | | |  |
| Study design | 4 | Present key elements of study design early in the paper | Dedicated section in M&M |
| Setting | 5 | Describe the setting, locations, and relevant dates, including periods of recruitment, exposure, follow-up, and data collection | Dedicated section in M&M |
| Participants | 6 | (*a*) Give the eligibility criteria, and the sources and methods of selection of participants. Describe methods of follow-up | Dedicated section in M&M |
|  |  | (*b*) For matched studies, give matching criteria and number of exposed and unexposed | None |
| Variables | 7 | Clearly define all outcomes, exposures, predictors, potential confounders, and effect modifiers. Give diagnostic criteria, if applicable | Dedicated section in M&M |
| Data sources/ measurement | 8* | For each variable of interest, give sources of data and details of methods of assessment (measurement). Describe comparability of assessment methods if there is more than one group | Dedicated section in M&M |
| Bias | 9 | Describe any efforts to address potential sources of bias | None |
| Study size | 10 | Explain how the study size was arrived at | Dedicated section in M&M |
| Quantitative variables | 11 | Explain how quantitative variables were handled in the analyses. If applicable, describe which groupings were chosen and why | Dedicated section in M&M |
| Statistical methods | 12 | (*a*) Describe all statistical methods, including those used to control for confounding | Dedicated section in M&M |
|  |  | (*b*) Describe any methods used to examine subgroups and interactions | Dedicated section in M&M |
|  |  | (*c*) Explain how missing data were addressed | None |
|  |  | (*d*) If applicable, explain how loss to follow-up was addressed | None |
|  |  | (*e*) Describe any sensitivity analyses | None |
| Results | | |  |
| Participants | 13* | (a) Report numbers of individuals at each stage of study—eg numbers potentially eligible, examined for eligibility, confirmed eligible, included in the study, completing follow-up, and analysed | Dedicated section in Results |
|  |  | (b) Give reasons for non-participation at each stage | None |
|  |  | (c) Consider use of a flow diagram | None |
| Descriptive data | 14* | (a) Give characteristics of study participants (eg demographic, clinical, social) and information on exposures and potential confounders | None |
|  |  | (b) Indicate number of participants with missing data for each variable of interest | None |
|  |  | (c) Summarise follow-up time (eg, average and total amount) | Dedicated section in Results |
| Outcome data | 15* | Report numbers of outcome events or summary measures over time | Dedicated section in Results and Table |
| Main results | 16 | (*a*) Give unadjusted estimates and, if applicable, confounder-adjusted estimates and their precision (eg, 95% confidence interval). Make clear which confounders were adjusted for and why they were included | Dedicated section in Results and Table |
|  |  | (*b*) Report category boundaries when continuous variables were categorized | None |
|  |  | (*c*) If relevant, consider translating estimates of relative risk into absolute risk for a meaningful time period | None |
| Other analyses | 17 | Report other analyses done—eg analyses of subgroups and interactions, and sensitivity analyses | Dedicated section in Results and Table |
| Discussion | | |  |
| Key results | 18 | Summarise key results with reference to study objectives | Followed |
| Limitations | 19 | Discuss limitations of the study, taking into account sources of potential bias or imprecision. Discuss both direction and magnitude of any potential bias | Followed |
| Interpretation | 20 | Give a cautious overall interpretation of results considering objectives, limitations, multiplicity of analyses, results from similar studies, and other relevant evidence | Followed |
| Generalisability | 21 | Discuss the generalisability (external validity) of the study results | Followed |
| Other information | | |  |
| Funding | 22 | Give the source of funding and the role of the funders for the present study and, if applicable, for the original study on which the present article is based | None |

*Give information separately for exposed and unexposed groups.

**Table S2.** The OHIP-14 questionnaire.

| **Domain Item** | **Fourteen questions** |
| --- | --- |
| Domain 1: Functional limitation | 1. Had trouble pronouncing any words  2. Felt sense of taste has worsened |
| Domain 2: Physical pain | 3. Had painful aching  4. Found it uncomfortable to eat any foods |
| Domain 3: Psychological discomfort | 5. Been self-conscious  6. Felt tense |
| Domain 4: Physical disability | 7. Felt diet has been unsatisfactory  8. Had to interrupt meals |
| Domain 5: Psychological disability | 9. Found it difficult to relax  10. Been a bit embarrassed |
| Domain 6: Social disability | 11. Been a bit irritable  12. Had difficulty doing usual jobs |
| Domain 7: Handicap | 13. Felt life less satisfying  14. Been totally unable to function |

Abbreviations: OHIP-14, Oral Health Impact Profile-14.
